# Six Weeks of Aerobic Exercise in Untrained Men With Overweight/Obesity Improved Training Adaptations, Performance and Body Composition Independent of Oat/Potato or Milk Based Protein-Carbohydrate Drink Supplementation

**DOI:** 10.3389/fnut.2021.617344

**Published:** 2021-02-15

**Authors:** Stefan Pettersson, Fredrik Edin, Carl Hjelte, David Scheinost, Sandro Wagner, Björn Ekblom, Niels Jessen, Klavs Madsen, Ulrika Andersson-Hall

**Affiliations:** ^1^Department of Food and Nutrition, and Sport Science, Centre for Health and Performance, University of Gothenburg, Gothenburg, Sweden; ^2^Department of Anesthesiology and Intensive Care Medicine, Sahlgrenska University Hospital, Gothenburg, Sweden; ^3^The Swedish School of Sport and Health Sciences, Stockholm, Sweden; ^4^Department of Biomedicine, Aarhus University, Aarhus, Denmark; ^5^The Norwegian School of Sports Sciences, Oslo, Norway; ^6^Institute of Neuroscience and Physiology, Sahlgrenska Academy, University of Gothenburg, Gothenburg, Sweden

**Keywords:** plant- and animal-based protein, endurance training, blood lipids and glucose, mitochondrial-related muscle proteins/enzymes, muscle biopsies

## Abstract

**Background:** Protein availability around aerobic exercise might benefit aerobic capacity and body composition in normal weight adults. However, it is unknown if individuals with overweight/obesity elicit similar adaptations or improve other cardiometabolic/health-related markers in response to different types of protein. Thus, our aim was to study the effect of supplementation of two different protein drinks in conjunction with exercise on aerobic capacity, body composition and blood health markers in untrained subjects with overweight or obesity.

**Methods:** The present study measured training adaptation and health parameters over a 6 week period in untrained men with overweight/obesity (*n* = 28; BMI 30.4 ± 2.2 kg/m^2^) ingesting either plant- (Oat/Potato; *n* = 8) or animal-based (Milk; *n* = 10) protein-carbohydrate drinks (10 g of protein/serving), or a control carbohydrate drink (*n* = 10) acutely before and after each training session (average three sessions/week @ 70% HR_max_). Pre-post intervention ${\overset{˙}{\text{V}}\text{O}}_{2\text{peak}}$, muscle biopsies and blood samples were collected, body composition measured (DXA) and two different exercise tests performed. Body weight was controlled with participants remaining weight stable throughout the intervention.

**Results:** For the groups combined, the training intervention significantly increased ${\overset{˙}{\text{V}}\text{O}}_{2\text{peak}}$ (8%; *P* < 0.001), performance in a time-to-exhaustion trial (~ 100%; *P* < 0.001), mitochondrial protein content and enzyme activity (~20–200%). Lean body mass increased (1%; *P* < 0.01) and fat mass decreased (3%; *P* < 0.01). No significant effects on fasting blood glucose, insulin, lipids or markers of immune function were observed. There were no significant interactions between drink conditions for training adaptation or blood measurements. For body composition, the Oat/Potato and carbohydrate group decreased leg fat mass significantly more than the Milk group (interaction *P* < 0.05).

**Conclusions:** Aerobic capacity and body composition were improved and a number of mitochondrial, glycolytic and oxidative skeletal muscle proteins and enzyme activities were upregulated by a 6 week training intervention. However, none of the parameters for endurance training adaptation were influenced by protein supplementation before and after each training session.

## Introduction

Overweight (BMI ≥ 25 kg/m^2^) and obesity (BMI ≥ 30 kg/m^2^) are defined as excess adiposity that presents a risk to health (1). High BMI has been associated with sedentary behavior (2), a decrease in relative fat free mass (FFM) (3) and low physical fitness and aerobic capacity (i.e., maximal oxygen uptake; ${\overset{˙}{\text{V}}\text{O}}_{2\text{max}}$) (4), factors which all have been linked to an increased risk of cardiometabolic diseases such as type 2 diabetes, cardiovascular diseases and dyslipidemia (3). Clearly, there is a need for interventions that can reverse the negative impacts on health and well-being inflicted by overweight/obesity.

Regular aerobic exercise is a well-proven strategy to increase ${\overset{˙}{\text{V}}\text{O}}_{2\text{max}}$ and endurance performance (5), changes underpinned by improved cardiac output and mitochondrial function and vascularity within skeletal muscle tissue (6). Other benefits of chronic aerobic exercise are changes in body composition and a positive influence on a range of other health markers, including lipid profile, the immune system, glucose and insulin metabolism (7–9).

In addition, the degree of exercise-induced adaptations can be influenced by nutritional factors such as protein intake (10). While the mechanisms and beneficial effects of post-resistance exercise protein supplementation on muscle protein synthesis and hypertrophy is well-researched, less is known about protein intake and endurance training adaptations. Dietary protein has been suggested to play a role on the synthesis of capillaries, synthesis and turnover of mitochondrial proteins and proteins involved in oxygen transport (11). Indeed, some research (12–14) but not all (15–18) has shown further improvements in ${\overset{˙}{\text{V}}\text{O}}_{2\text{max}}$ and increased FFM (12, 13, 17) following 4.5–12 weeks of post-exercise protein supplementation. Moreover, while markers of mitochondrial adaptation such as citrate synthase (CS), succinate dehydrogenase (SDH) activity and PGC-1α protein content has been shown to be increased by the training intervention *per se*, this increment appears to be independent of post-exercise protein or carbohydrate only intake (12, 13). However, it is important to note that all previous aerobic training interventions investigating responses of protein supplementation on physiological adaptations has included healthy, recreationally active or well-trained participants with normal body weight (BMI ≤ 25 kg/m^2^). High BMI has been related to an increment in post-exercise muscle damage/CK-levels (19), and obesity has both been shown to interfere with physiological changes occurring in the mitochondria in response to acute elevation in the plasma amino acids (20), and to be associated with a blunted myofibrillar protein synthetic response to dietary protein ingestion following resistance exercise (21). Thus, considering that it currently is unknown if chronic protein supplementation in conjunction with aerobic exercise results in similar responses in overweight/obese individuals as in normal weight individuals, and that improvements in ${\overset{˙}{\text{V}}\text{O}}_{2\text{max}}$ and FFM within the former population can have positive effects extending beyond performance enhancement (i.e., health benefits), warrants further research.

Acute exercise studies suggests that the protein source might influence the degree of the anabolic response due to differences in amino acid and peptide profile, protein digestion and absorption kinetics (22). A number of studies have demonstrated that milk (whey and/or casein) and other animal-based protein sources (egg, beef) are superior to plant based-proteins in muscle anabolism in young and elderly healthy individuals (23) although Oikawa et al. (24) demonstrated that potato protein isolate, which has a protein quality/essential amino acid content similar to milk-based proteins (25), also have the ability to stimulate muscle protein synthesis at rest and with resistance exercise. Also, a meta-analysis showed that supplementing ≥6 weeks resistance training programs with soy or whey protein produces similar gains in strength and lean body mass (26). Besides soy protein, few studies have investigated the effects of other plant-based protein sources in conjunction with training (27, 28). Babault et al. (27) reported similar gains in upper limb muscle thickness and strength following daily supplementation of a pea protein isolate or a whey protein concentrate in young healthy males performing a 12-week resistance training protocol. In an 8-week training intervention, post-resistance exercise supplementation of both whey and rice protein isolate improved indices of body composition and performance in college-aged men, but no differences between the two groups were observed (28). However, to our knowledge, no previous research has investigated the long-term effects of plant- vs. animal-based protein supplementation with aerobic exercise.

The present study aimed to compare the intake of a potato protein supplemented oat carbohydrate drink with a milk protein and carbohydrate drink and a control carbohydrate drink, before and after aerobic exercise, during a randomized controlled 6 weeks intervention in untrained men with BMI 28–32 kg/m^2^. Based on previous research with similar study design and training status of the participants (12), we hypothesized that, compared to carbohydrate-only intake, protein supplementation would improve endurance parameters such as ${\overset{˙}{\text{V}}\text{O}}_{2\text{peak}}$. Considering the resemblance in amino acid composition of the plant- and milk-based protein drinks in the current study, we expected these improvements irrespective of type of protein source. Thus, the primary outcome was the effect on ${\overset{˙}{\text{V}}\text{O}}_{2\text{peak}}$. Secondary outcomes were other effects on training adaptations (e.g., activity and expression of skeletal muscle enzymes and proteins involved in glycolytic or oxidative metabolism and recovery CK levels), performance, body composition and the effect on parameters associated with disease (fasting glucose and blood lipids, insulin/insulin resistance (HOMA-IR) and immune markers (TNF-alpha, IL-6 and hs-CRP).

## Subjects and Methods

### Study Participants

Inclusion criteria were non-smoking men with body mass index 28–32 kg/m^2^ and between 20 and 40 years of age with no chronic disease. Participants were recruited locally from the University campus or central Gothenburg area, Sweden, by posters and advertisements. A screening before participation was undertaken to ensure they were free from any medical condition that would preclude their participation in the study. Exclusion criteria for participation included food allergies, adherence to non-traditional dietary practices such as low carbohydrate or ketogenic diets, and use of medication known to influence lipid metabolism. Furthermore, participants should not have engaged in conditioning exercises > 2 h/week during the 6 months prior to study initiation. We initially recruited 30 participants but had two drop-outs of the study for reasons unrelated to the study (both from the Oat/Potato group). Consequently, 28 men with BMI 30.4 ± 2.2 kg/m^2^, age 28.4 ± 5.2 years participated in the study. Thirteen participants were overweight (BMI > 25 kg/m^2^) and fifteen were obese (BMI > 30 kg/m^2^). The project was performed in accordance with the Declaration of Helsinki 2008 and approved by the local ethical committee in Gothenburg (Dnr 571-14) and all participants gave their written informed consent.

### Study Design

**Figure 1:**
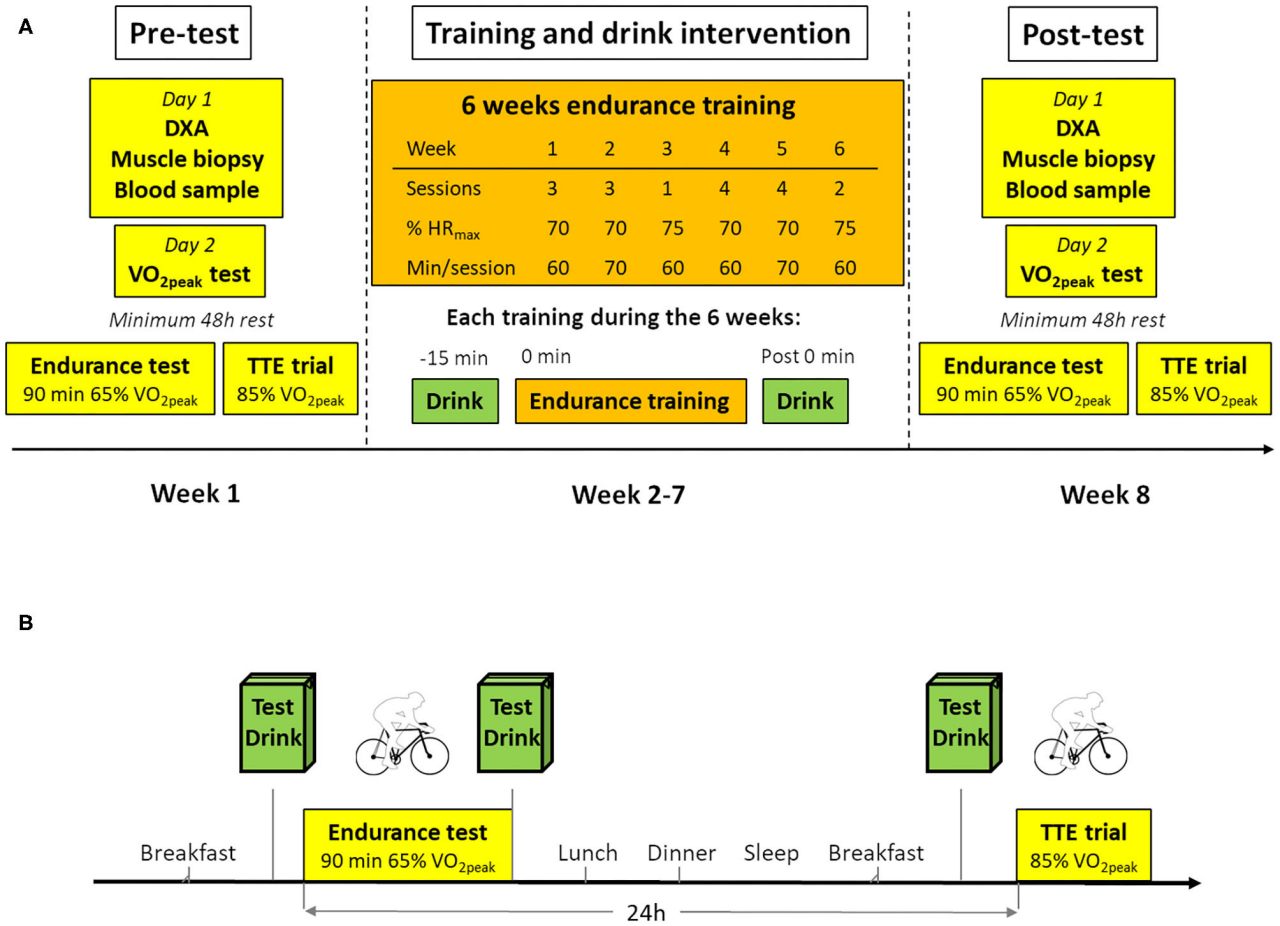
Study Design. **(A)** Overview of the study including 6 weeks intervention and tests and subject characterization before and after the intervention. **(B)** Overview of the endurance and recovery tests performed before and after the intervention. Details of tests and training are outlined in methods section. TTE trial, Time to exhaustion test.

The 8-week study protocol had a randomized parallel group design (Figure 1A). The experimental protocol consisted of baseline measurements (Pre-test; week 1), where participants were required to attend four separate-day visits to the laboratory and to complete a 3-day dietary food record. Assessment included body composition, a fasting blood sample, a muscle biopsy sample with all three assessments conducted on the same day in that order, a cardiorespiratory fitness test [${\overset{˙}{\text{V}}\text{O}}_{2\text{peak}}$), an endurance test and a performance/time to exhaustion test (TTE)]. The ${\overset{˙}{\text{V}}\text{O}}_{2\text{peak}}$ test was performed on a separate day allowing for a minimum of a two-day rest before the endurance test. All exercise tests were performed on the same cycle ergometer (Monark 828 E, Varberg, Sweden) with the same single-blinded test leader. All participants and the staff performing body composition measurements, overseeing the training sessions and analyzing the data were double-blinded. For the 3-day food record, participants received written and verbal instructions on registering food and fluid intake. They were instructed to document all consumed foods and fluids, and fat percentage of dairy products in the food and fluid diary. The quantity of all food items was reported in household measures or, if available, by packaging details. The food records were analyzed for macronutrient composition and total caloric intake (Supplementary Table) using the nutrient calculation software package *Dietist XP* version 3.2 (Kost & Näringsdata, Sweden), which references the Swedish Food Composition Database (Swedish National Food Agency 2013–10–04). The training intervention (week 2–7) consisted of 1–4 days/week of supervised endurance exercise training as described below. During week 8 (Post-test), the Pre-test protocol was repeated in the same way as during week 1. Participants were randomly assigned to one of three BMI-matched groups that defined their pre- and post-exercise drink intake: Oat/Potato, Milk, or Control (Table 1). Since body weight loss can be a confounding factor to variables of interest in the present study, the participants were advised to remain weight stable and were given nutritional counseling (increased carbohydrate and fat intake) during the intervention if they deviated ≥ 2% from initial weight.

**Table 1:** Subject characteristics and training data.

	**Control (*n* = 10) Mean ± SD**	**Oat/Potato (*n* = 8) Mean ± SD**	**Milk (*n* = 10) Mean ± SD**
Age (years)	27.9 ± 5.0	29.3 ± 5.8	28.2 ± 5.5
BMI (kg/m^2^)	30.4 ± 1.8	31.3 ± 2.3	29.8 ± 2.3
${\overset{˙}{\text{V}}\text{O}}_{2\text{peak}}$ (L/min)	3.8 ± 0.6	4.1 ± 0.7	3.9 ± 0.6
W_max_ (Watt)	241 ± 54	269 ± 53	256 ± 50
**Supervised training sessions:**			
Compliance (% attendance)	100	99.4 ± 1.9	100
Total exercise time (min/subject)	1,109 ± 42	1,090 ± 0	1,102 ± 49
Mean intensity (% of HR_max_)	71 ± 1	70 ± 1	70 ± 1

### Test Drinks

The nutritional content and amino acid composition of the three test drinks is presented in Table 2. The Oat/Potato drink, which was a prototype of a vegan protein drink consisted primarily of an (11.5% dry matter) oat base (166 g/250 ml serving) plus water (69 g). The protein content of the oat base (about 1% inherent oat protein) was supplemented with 6 plus 3 g of potato protein isolate per serving (Solanic 300XP and Solanic 300N, respectively, AVEBE, Veendam, the Netherlands) to match the amino acid composition of the milk drink. Each 250 ml serving of the Oat/Potato drink contained 1.25 g β-glucans from the oatbase and 0.25 g of PromOat β-glucan (Tate & Lyle Oat, Kimstad, Sweden). Further, 2 g rapeseed oil were added to each serving. The main ingredients in the milk drink (per 250 ml serving) was low lactose, 1.5% fat dairy milk (243 g) with the addition of rapeseed oil (1.2 g), whey protein (0.4 g; WPC 80, Wheyco, Hamburg, Germany), Na-caseinate (1.5 g; Miprodan 30, Arla Foods Amba) and 4 g dextrose to match the total fat/protein/carbohydrate content of the Oat/Potato drink The control drink consisted of 10, 5 and 7.6 g of maltodextrin, dextrose and rapeseed oil, respectively, mixed in 226 g of water. For blinding purposes, vanilla aroma was included in all three drinks which were produced and packed at the same facility (TetraPak, Lund, Sweden). The nutritional composition of the Oat/Potato, Milk and Control drinks were analyzed by an independent, accredited laboratory (Eurofins Food & Feed Testing, Linköping, Sweden).

**Table 2:** Contents of test drinks (250 ml).

	**Control**	**Oat/Potato**	**Milk**
Energy content (kcal)	134	140	127
Carbohydrate (g)	15	16	15
Fat (g)	7.6	3.8	3.2
β-glucans (g)	-	1.5	-
Protein (g)	-	10	9
Amino acid content (g)			
Alanine	-	0.3	0.3
Arginine	-	0.5	0.3
Aspartic acid	-	1.3	0.7
Cysteine + Cystine	-	0.3	0.1
Phenylalanine	-	0.7	0.4
Glutamic acid	-	1.1	2.0
Glycine	-	0.6	0.2
Histidine	-	0.2	0.2
Isoleucine	-	0.5	0.5
Leucine	-	1.0	0.9
Lysine	-	0.7	0.7
Methionine	-	0.1	0.2
Proline	-	0.6	0.9
Serine	-	0.6	0.5
Threonine	-	0.5	0.4
Tyrosine	-	0.5	0.4
Valine	-	0.8	0.6
Tryptophan	-	0.1	0.1

### Body Composition and Blood Sampling

Following an overnight fast, participants were weighed in their underwear and body composition was assessed by dual-energy X-ray absorptiometry (iDXA; GE Medical Systems, Madison, WI, USA). The DXA was calibrated according to the manufacturer's guidelines before each measurement. Total-body and regional lean and fat mass analysis was automatically made by the enCore software (version 16.10). CV for duplicate measurements were 0.69% for body fat, 0.71 kg for fat mass and 0.76 kg for lean mass. Venous blood was drawn in vacutainer EDTA and serum separation tubes. Serum was centrifuged at 1,800 g for 10 min and EDTA plasma at 2,200 g for 15 min at 4°C. Plasma and serum were immediately aliquoted and stored at −80°C.

### Skeletal Muscle Biopsies

Skeletal muscle biopsies were obtained from the *m. vastus lateralis* under local anesthesia (1% lidocaine) of the skin and superficial muscle fascia with the use of a conchotome according to the technique of Dietrichson et al. (29). The post-intervention biopsy was taken 4 ± 3 days after the last training session with no difference between groups regarding timing of biopsy. The biopsy was immediately dissected to be free of fat and connective tissue and divided into two parts for later determination of enzyme capacity (15 mg) and Western blotting (40 mg). The two parts were immediately placed in liquid nitrogen and subsequently stored at −80°C until processing.

### Cardiorespiratory Fitness Test

The ${\overset{˙}{\text{V}}\text{O}}_{2\text{peak}}$ test was performed in a fed and rested state. Participants performed four 5-min steady state work-loads (105, 140, 175, and 70W) at a pre-determined cadence of 70 RPM, followed by a 17.5 W incremental increase of load (beginning at 200 W), followed by incremental increase of load every minute until exhaustion (e.g., failure to maintain the cadence). Rating of perceived exertion [RPE; Borg category scale 6–20 (30)] was recorded, and a fingertip blood sample (glucose and lactate) was collected at the end of each 5 min workload and following exhaustion. Heart rate (Polar M400 Polar Electro OY, Kempele, Finland) was continuously recorded, and pulmonary gas exchange (${\dot{\text{V}}\text{O}}_{2}$ and ${\dot{\text{V}}\text{CO}}_{2}$) was measured using a metabolic cart (Jaeger Oxycon Pro, Viasys Healthcare, Germany).

### Endurance and Performance Test

Following the ${\overset{˙}{\text{V}}\text{O}}_{2\text{peak}}$ test, participants were asked to refrain from vigorous physical activity for the 2 days before the experimental trials and pre- post-tests were performed at the same time in the morning to avoid circadian variance. The day before the endurance test participants were fitted with a continuous glucose monitoring system (CGM; Medtronic Paradigm Veo 554) and provided with a 200 ml liquid supplement (Fresubin Jucy Drink, Fresenius Kabi, Uppsala, Sweden) along with instructions to consume the drink at home as their only breakfast (05.45) i.e., 180 min before the onset of exercise (08.45) the following morning. The drink provided 8, 67, 0 and 0 g of protein, carbohydrates, fat and fiber, respectably. Compliance to drink ingestion was confirmed by the CGM system upon arrival to the laboratory.

The participants consumed 250 ml test drink (Oat/Potato, Milk and Control) 15 min before onset of exercise (5 min warm-up cycling at 55% ${\overset{˙}{\text{V}}\text{O}}_{2\text{peak}}$ followed by 90 min at 65% ${\overset{˙}{\text{V}}\text{O}}_{2\text{peak}}$, all at 70 RPM, all based on ${\overset{˙}{\text{V}}\text{O}}_{2\text{peak}}$ and W_max_ before the intervention) and 250 ml directly after (Figure 1B). Pulmonary gas exchange and heart rate were measured for 2 min at 15 min intervals throughout the test. Venous blood samples were collected 5-min before the first drink ingestion, directly after exercise and 60 and 120 min post-exercise. Before leaving the laboratory, participants were served lunch and received pre-packed take-home meals (dinner, 2 × afternoon/evening snack and breakfast-drink), all calculated based on their estimated daily energy requirements. The meals were instructed to be eaten with 3-h intervals following lunch until bedtime. The Mifflin-St Jeor equation with a standardized activity factor of 1.4 plus energy expenditure from indirect calorimetry (90 min at 65% ${\overset{˙}{\text{V}}\text{O}}_{2\text{peak}}$) was used to estimate daily energy requirements.

To investigate the acute recovery effect of the tested drinks, a performance test of 85% ${\overset{˙}{\text{V}}\text{O}}_{2\text{peak}}$ until exhaustion (TTE) was performed 24-h after the endurance test. Similar to test day 1, the 200 ml liquid supplement (breakfast) and pre-test drink (Oat/Potato, Milk or Control) were consumed 180 and 15 min before beginning TTE, respectively. The TTE started with a warm-up (5 min at 60% ${\overset{˙}{\text{V}}\text{O}}_{2\text{peak}}$ and 5 min at 70% ${\overset{˙}{\text{V}}\text{O}}_{2\text{peak}}$), after which the power was increased to 85% of ${\overset{˙}{\text{V}}\text{O}}_{2\text{peak}}$ and the timer started. Participants were instructed to ride until exhaustion, which was defined as failure to maintain the pre-determined cadence (70 rpm) and were verbally encouraged throughout. At the second warning of failing to maintain the cadence, the trial was terminated and time to exhaustion was recorded. The bike and test leader was the same for all test. Blood samples were collected before test drink ingestion, directly after exercise and a venous sample after a recovery period of 30 min.

### Training Intervention

All participants performed 6 weeks of supervised physical training (Table 1). The participants were offered time-slots between 8.30 a.m. and 19.00 p.m. all weekdays, with the majority of training sessions taking place in the afternoon (14–19.00 p.m.). The participants were instructed to refrain from energy containing food/fluid intake 1 and 2 h before and after exercise, respectively. At each training session, the test drink was administered 15 min pre- and immediately post-exercise. In order to increase compliance/reduce potential dropouts the participants were free to choose between treadmill running (RL2500E, Rodby Innovation, Vänge, Sweden), rowing (Concept 2, Morrisville, USA) or bike exercise (Monark 828 E, Varberg, Sweden) contingent that a minimum of 20 min per session was spent on each exercise modality of choice. There was no difference between groups in time spent on each exercise modality (*P* > 0.4, mean time was 58% cycling, 19% rowing and 23% running). The training load in each training session was aimed at 70–75% of HR_max_ and the time ranging from 60 to 75 min as stated in Figure 1. Excluding the 5 min warmup, heart rate was monitored continuously throughout training sessions. In order to avoid overload, the training was planned with a pattern of two moderate to hard weeks (3 sessions per week) followed by a week with less training (1 session), repeated with another 2 hard weeks (4 sessions per week) and finished with a lighter week (2 sessions). Compliance to the training program was 99.8% of the planned training, and the mean training load did not differ between groups (Table 1).

### Blood Analysis

Serum creatine kinase (CK) concentrations were analyzed by a spectrophotometric method according to a previously described method (31). IL-6 and TNF-α were assayed using ELISA kits from R&D Systems, Minneapolis, MN, USA (catalog No. HS600B and HSTA00C, respectably). Variation coefficient (CV) for CK, IL-6 and TNF-α is 3–4, 9, and 16%, respectively (31, 32). Glucose, insulin, cholesterol (total, low-density lipoprotein [LDL], high-density lipoprotein [HDL]), triglycerides (TG) and C-reactive protein (CRP) were routinely analyzed at the accredited Clinical Chemistry Laboratory, Sahlgrenska University Hospital (International Standard ISO 15189:2007) using a Cobas Modular system (Roche Diagnostics, Risch, Switzerland). CV for insulin was 10%, TG 4%, cholesterol 3%, CRP 3–4% and glucose 3%. Homeostatic model of insulin resistance (HOMA-IR) was calculated as (fasting glucose × fasting insulin)/22.5 (33). Two fasting blood samples were excluded from analysis due to participants not complying with the fasting protocol.

### Skeletal Muscle Enzyme Activity

One portion of powdered muscle was homogenized on ice in phosphate buffer (1 mg dry weight per 0.400 ml) and used for CS, HAD and LDH determination. The maximal activity of the enzymes were determined fluorometrically (Fluorometer 810) by measuring NADH after adding acetyl-CoA (CS) or acetoacetyl-CoA (HAD) according to previously described methods (34).

### Skeletal Muscle Protein Extraction and Western Blot Analysis

Frozen muscle biopsies were freeze-dried 48 h in a Heto Drywinner (Heto Holten A/S, Allerød, DK) at −96°C and 0.5 mmHg. Freeze-dried muscle tissue were homogenized in ice-cold lysis buffer (50 mM HEPES, 137 mM NaCl, 10 mM Na_4_P_2_O_7_, 20 mM NaF, 5 mM EDTA, 1 mM MgCl, 1 mM CaCl_2_, 2 mM Na_3_OV_4_, 5 mM Nicotinaminde, 10 μM Trichostatin A, 1% (vol/vol) HALT protease inhibitor cocktail, 1% (vol/vol), NP-40, 10% (vol/vol) glycerol) using a Precellys homogenizer (Bertin Technologies, FR). Insoluble materials were removed by centrifugation at 14,000 × g for 20 min at 4°C. Protein concentration of the supernatant was determined using a Bradford assay (BioRad, CA, USA). Samples were adjusted to equal concentrations with milli-Q water and denatured by mixing with 4 × Laemmli's buffer and heating at 95°C for 5 min. Equal amounts of protein (30 μg in each well) were separated by SDS-PAGE using the BioRad Criterion system, and proteins were electroblotted onto PVDF membranes (BioRad). Control for equal loading was performed using the Stain-Free technology (35, 36). Membranes were blocked for 2 h in a 2% bovine serum albumin solution (Sigma-Aldrich, MO, USA) and incubated overnight with primary antibodies against GLUT4 (#07-1404) and IRS-1 (#06-248) from Millipore, cytochrome C (#4272), PDH (#3205), Hexokinase II (#2867), glycogen synthase (#3886), Hexokinase II (#2867) SDHA (#11998), and IR (#3025) all from Cell Signaling. After incubation in primary antibodies the membranes were washed three times and then incubated 1 h with HRP-conjugated secondary antibodies. Proteins were visualized by chemiluminiscence (Pierce Supersignal West Dura, Thermo Scientific, IL, USA) and quantified with ChemiDoc^TM^ MP imaging system (BioRad). Calibration curves have been used during validation of the antibodies, but was not included in the gels for this experiments. The concentrations were expressed as % of the mean for the whole population pre-intervention. For HK-II, the lower band was quantified and for PDH the whole band from upper to lower pole was quantified. Protein Plus Precision All Blue standards were used as markers of molecular weight (BioRad).

### Statistical Analysis

#### Power Calculation

Sample size (n) was estimated via G^*^ software (Version 3.1.9.2, Universität Düsseldorf, Germany) using the Fixed effects, omnibus, one-way ANOVA setting. The calculation was based on previous research by Ferguson-Stegall et al. (12) demonstrating a significant increase in ${\overset{˙}{\text{V}}\text{O}}_{2\text{max}}$ following 4.5 weeks of protein supplementation compared to carbohydrate-only supplementation in untrained participants, and *n* = 8 participants was determined to be adequate to detect differences in mean pre-post ${\overset{˙}{\text{V}}\text{O}}_{2\text{peak}}$ values between groups, assuming a power of 80% and an α-level of 5%.

#### General Statistics

All exercise and DXA data, and the majority of blood and muscle measurements, fulfilled the criteria for normality using Shapiro–Wilk testing. On inspection, normality and residual plots for all other variables were deemed satisfactory apart from insulin, HOMA-IR and CRP. The logarithms for insulin, HOMA-IR and CRP were, however, normally distributed and thereby used for further analysis. One-way ANOVA analysis was used to compare baseline data between the three randomized groups. With the exception of IL-6, no pre-test differences between the groups in any primary or secondary outcome variables were observed. For between-group comparisons, two-way repeated measures ANOVA (treatment × time) before and after 6 weeks of exercise training and Oat/Potato, Milk or Control intervention was used in order to compare changes in ${\overset{˙}{\text{V}}\text{O}}_{2\text{peak}}$, performance, recovery, body composition, blood values, muscle enzymes and proteins. A two-way repeated measures ANOVA (treatment × time) was also performed for the two protein interventions combined vs. the Carbohydrate control group. In addition, for the time course, a three-way repeated measures ANOVA (treatment × time pre/post × time course for sampling) was used in order to compare changes for CK. Tukey *post-hoc* analysis was performed when a significant group differences or interactions occurred in the ANOVA test. Partial Eta-squared (_p_η^2^) was calculated as a measure of effect size for the ANOVA, where values of 0.01, 0.06, and 0.15 were considered as small, medium, and large, respectively (37). All analysis was performed using SPSS version 27 (IBM SPSS Statistics, USA). *P* < 0.05 was considered significant. Values are expressed as mean ± SD in text and tables, and mean ± SE in graphs.

## Results

### Aerobic Capacity

${\overset{˙}{\text{V}}\text{O}}_{2\text{peak}}$ increased following the 6 weeks exercise training in all groups (Table 3). There was no significant effect of the drink interventions, where all three groups obtained similar increases of around 6–8% in ${\overset{˙}{\text{V}}\text{O}}_{2\text{peak}}$. Data from the endurance tests before and after intervention showed a similar and significant training effect in all groups with a lower $\dot{\text{V}}$O2, heart rate, lactate and RER after the intervention at the defined power output (Table 4). There were no significant differences between the three drink interventions.

**Table 3:** ${\overset{˙}{\text{V}}\text{O}}_{2\text{peak}}$ and recovery performance test before and after the intervention.

		**Control (*n* = 10)**	**Oat/Potato (*n* = 8)**	**Milk (*n* = 10)**	**Two-way ANOVA** ***P*****-value (**_****p****_*****η*****^****2****^**)**		
		**Mean ± SD**	**Mean ± SD**	**Mean ± SD**	**Time**	**Group**	**Interaction**
${\overset{˙}{\text{V}}\text{O}}_{2\text{peak}}$ (ml/min/kg)	Pre	38.2 ± 5.9	39.7 ± 4.7	38.6 ± 5.6	**<0.001** (0.709)	0.798 (0.018)	0.341 (0.082)
	Post	41.5 ± 5.4	43.0 ± 4.0	40.8 ± 6.8			
Performance TTE (min)	Pre	19.3 ± 9.2	22.7 ± 11.9	11.4 ± 3.6	**<0.001** (0.604)	**0.035** (0.243)^*^	0.306 (0.094)
	Post	56.1 ± 35.2	56.2 ± 20.7	30.6 ± 20.3			

* *Milk significantly different to both Control and Oat/Potato (Tukey post-hoc analysis). Significant P-value (<0.05) is displayed in bold. ANOVA, analysis of variance; _p_*η^2^*, partial Eta-squared*.

**Table 4:** Endurance test data before and after intervention.

		**Control (*n* = 10)**	**Oat/Potato (*n* = 8)**	**Milk (*n* = 10)**	**Two-way ANOVA *P*-value (_p_η^2^)**		
		**Mean ± SD**	**Mean ± SD**	**Mean ± SD**	**Time**	**Group**	**Interaction**
Lactate (mmol/L)	Pre	4.95 ± 1.63	4.22 ± 1.47	5.44 ± 2.50	**<0.001** (0.687)	0.565 (0.048)	0.484 (0.061)
	Post	2.49 ± 0.87	2.57 ± 1.45	2.88 ± 1.36			
${\dot{\text{V}}\text{O}}_{2}$ (L/min)	Pre	2.62 ± 0.37	2.88 ± 0.42	2.78 ± 0.39	**<0.001** (0.635)	0.416 (0.068)	0.835 (0.014)
	Post	2.47 ± 0.42	2.71 ± 0.40	2.64 ± 0.42			
HR (bpm)	Pre	156 ± 17	157 ± 10	160 ± 12	**<0.001** (0.580)	0.596 (0.041)	0.847 (0.013)
	Post	134 ± 11	140 ± 12	141 ± 20			
RER	Pre	0.93 ± 0.02	0.92 ± 0.03	0.94 ± 0.03	**<0.001** (0.391)	0.543 (0.048)	0.824 (0.015)
	Post	0.91 ± 0.02	0.90 ± 0.02	0.91 ± 0.04			

*Post-exercise lactate and average exercise ${\dot{\text{V}}\text{O}}_{2}$, heart rate and RER*.

*P-value (< 0.05) is displayed in bold. ANOVA, analysis of variance; _p_*η^2^*, partial Eta-squared*.

### Recovery Tests

At the TTE test before the training intervention, the Milk group cycled a significantly shorter time than the other two groups (Table 3). The training intervention had a large effect on TTE in all groups, with all participants more than doubling their time to exhaustion. There was no significant difference between groups in improvement following the 6 weeks of intervention.

**Figure 2:**
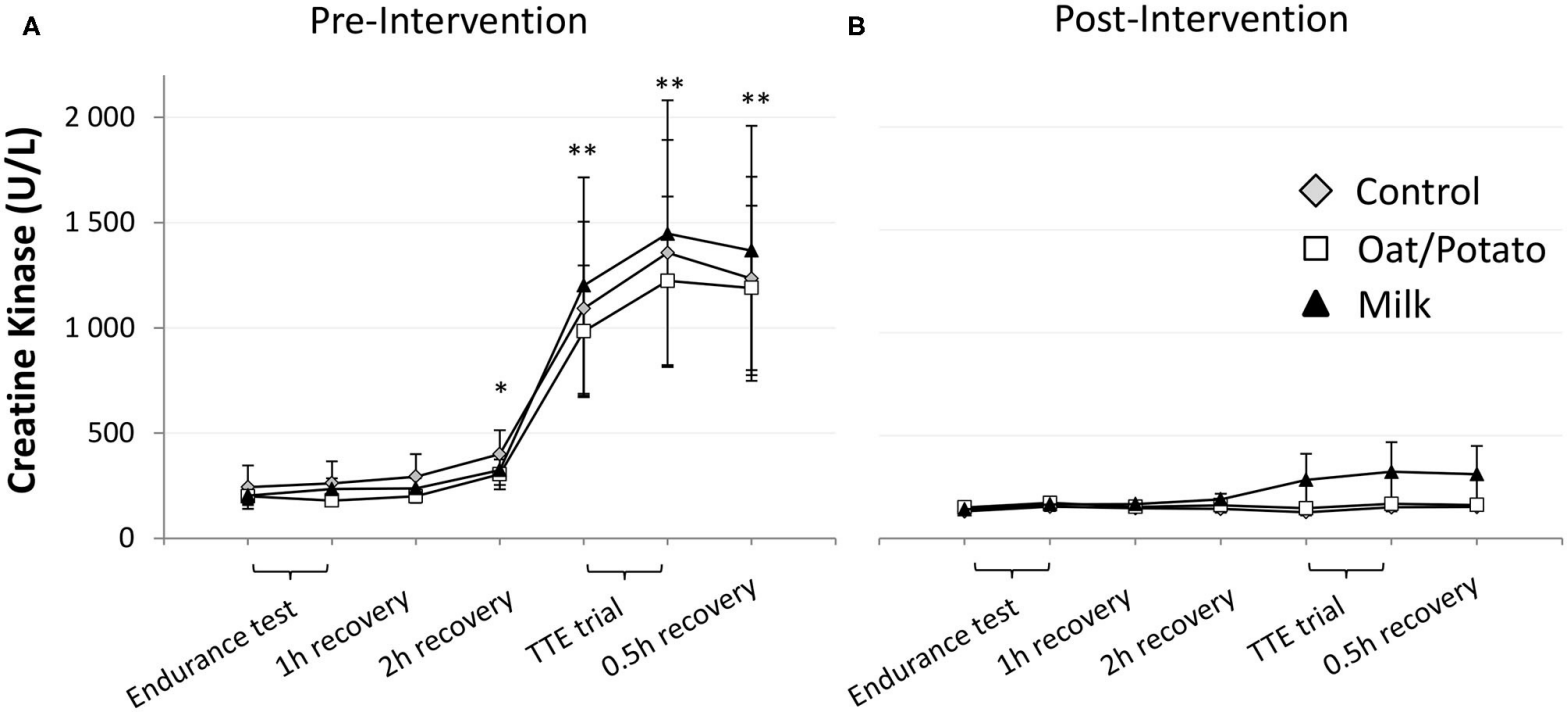
Serum creatine kinase (CK) levels during a 36 h period including the 90 min endurance test and next day TTE trial. This was done **(A)** pre and **(B)** post the 6 weeks training and drink (Control, Oat /Potato or Milk) intervention. Values are displayed as mean ± SE. *P < 0.05 and **P < 0.01 compared with starting values (all groups, pre-intervention). TTE, time to exhaustion.

Creatine kinase (CK) as a marker of muscle damage showed a marked and significant increase in all groups after the endurance test, starting at 2 h post-exercise and increasing to 1,200–1,500 U/L after 24 h (Figure 2A). The pattern was significantly affected by the 6 weeks training intervention (*P* < 0.001, three-way ANOVA) showing unaltered CK levels following the endurance test after the intervention in all three groups (Figure 2B). There were no differences in CK levels between the drink interventions before or after the 6 weeks training period.

### Muscle Enzyme Activities

**Figure 3:**
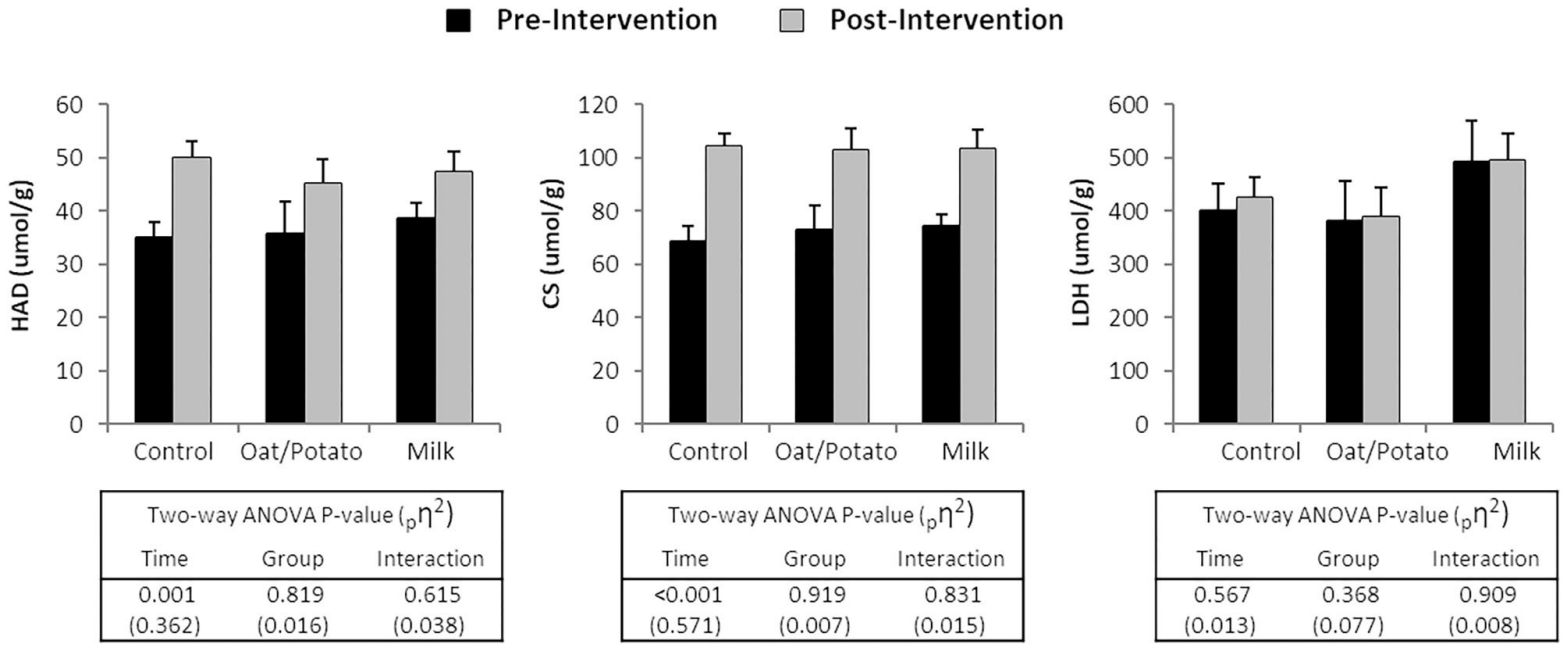
Enzyme activities of 3-OH-acyl-coA dehydrogenase (HAD), citrate synthase (CS) and lactate dehydrogenase (LDH) in muscle biopsies taken at rest before and after a 6 weeks training and drink (Control, Oat/Potato or Milk) intervention. Values are displayed as mean ± SE. ANOVA, analysis of variance; _p_η^2^, partial Eta-squared.

In resting muscle biopsies taken before and after the intervention, there was an increase in enzyme activities of oxidative enzymes CS and HAD in the whole group (*P* < 0.001) but no significant difference between the drink intervention groups (Figure 3). The glycolytic enzyme LDH did not change pre-post intervention.

### Muscle Protein Expression

**Figure 4:**
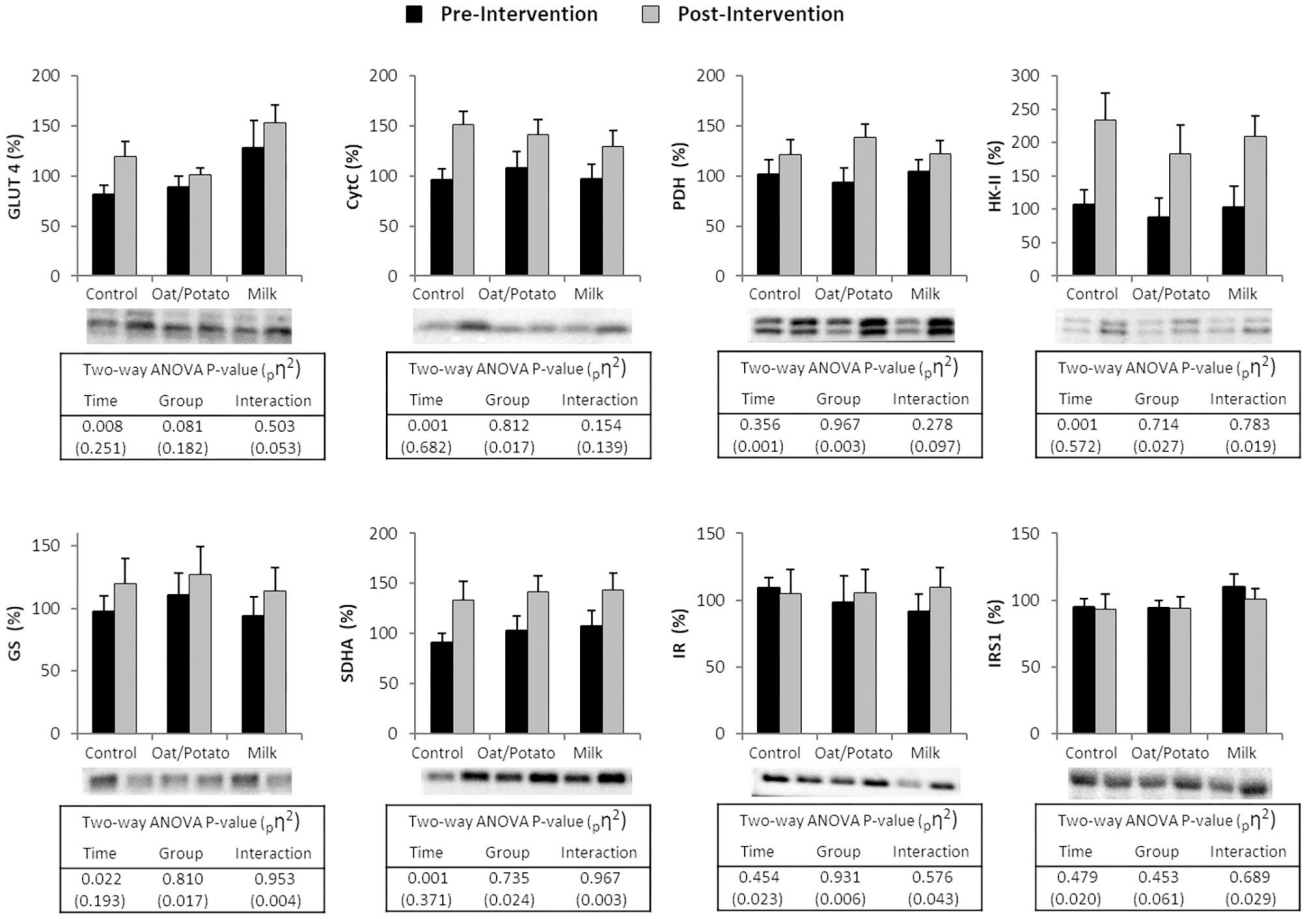
Western blot protein levels of GLUT4. Cytochrome C (CytC). Pyruvate Dehydrogenase (PDH). Hexokinase-II (HK-II). Glycogen Synthase (GS). Succinate Dehydrogenase subunit A (SDHA). Insulin Receptor (IR) and Insulin Receptor Substrate 1 (IRS1) in muscle biopsies taken at rest before and after a 6 weeks training and drink (Control, Oat/Potato or Milk) intervention. For each protein, the individual results have been standardized by the same coefficient where 100% represents the combined pre-intervention mean for all subjects. Values are displayed as mean ± SE, images show representative immunoblots. Representative blots ANOVA, analysis of variance; _p_η^2^, partial Eta-squared.

The expression of selected proteins are presented in Figure 4. The proteins that significantly increased during the intervention for the whole population were GLUT4, CytC, PDH, HK-II, SDHA and GS (*P* < 0.05). There were no significant differences between groups. IR and IRS1 levels were not changed pre-post intervention.

### Body Composition

Since weight loss *per se* can influence immune function, blood lipids and glucose metabolism, the participants received dietary advice with the intention to keep weight stable. Indeed, the weight was not significantly affected over the training intervention (Table 5), though there were changes in body composition. Total Lean body mass increased (*P* < 0.01) and fat mass decreased (*P* < 0.01) with no significant differences between the three drink interventions observed. A significant treatment × time interaction was evident in regional fat mass, with leg fat mass decreasing in Control and Oat/Potato but not in Milk [Table 6; interaction effect; *F*_(2,25)_ = 4.94; *P* < 0.05].

**Table 5:** Body composition before and after the intervention.

		**Control (*n* = 10)**	**Oat/Potato (*n* = 8)**	**Milk (*n* = 10)**	**Two-way ANOVA *P*-value (_p_η^2^)**		
		**Mean ± SD**	**Mean ± SD**	**Mean ± SD**	**Time**	**Group**	**Interaction**
Body Weight (kg)	Pre	100.1 ± 11.3	104.7 ± 10.8	101.7 ± 10.4	0.301 (0.043)	0.443 (0.063)	0.422 (0.067)
	Post	99.5 ± 10.8	103.9 ± 10.8	101.6 ± 10.5			
Lean Mass (kg)	Pre	63.2 ± 6.9	65.2 ± 6.5	64.1 ± 8.8	**0.002** (0.321)	0.859 (0.012)	0.769 (0.021)
	Post	63.8 ± 6.4	65.7 ± 6.2	65.0 ± 8.7			
Fat Mass (kg)	Pre	32.9 ± 8.3	34.4 ± 6.9	32.4 ± 6.9	**0.002** (0.322)	0.880 (0.010)	0.116 (0.158)
	Post	31.4 ± 8.3	33.3 ± 7.0	32.3 ± 8.0			
Body Fat (%)	Pre	32.8 ± 5.8	33.3 ± 4.7	32.4 ± 5.9	**<0.001** (0.411)	0.966 (0.003)	0.305 (0.091)
	Post	31.6 ± 5.9	32.3 ± 4.8	32.0 ± 6.7			

*Significant P-value (<0.05) is displayed in bold. ANOVA, analysis of variance; _p_*η^2^*, partial Eta-squared*.

**Table 6:** Regional body composition measures before and after the intervention.

		**Control (*n* = 10)**	**Oat/Potato (*n* = 8)**	**Milk (*n* = 10)**	**Two-way ANOVA *P*-value (_p_η^2^)**		
		**Mean ± SD**	**Mean ± SD**	**Mean ± SD**	**Time**	**Group**	**Interaction**
**Lean mass**							
Arms (kg)	Pre	8.0 ± 1.2	8.4 ± 1.4	8.4 ± 1.3	0.346 (0.036)	0.680 (0.030)	0.866 (0.011)
	Post	7.9 ± 1.1	8.4 ± 1.4	8.4 ± 1.3			
Legs (kg)	Pre	22.9 ± 2.8	24.1 ± 2.3	23.5 ± 3.7	**0.001** (0.376)	0.704 (0.028)	0.959 (0.003)
	Post	23.2 ± 2.5	24.4 ± 2.1	23.9 ± 3.6			
Trunk (kg)	Pre	28.3 ± 3.2	28.9 ± 3.2	28.5 ± 3.9	**0.006** (0.266)	0.971 (0.002)	0.635 (0.036)
	Post	28.9 ± 3.0	29.1 ± 3.3	29.0 ± 3.9			
**Fat mass**							
Arms (kg)	Pre	3.2 ± 0.7	3.5 ± 0.9	3.2 ± 0.9	0.116 (0.096)	0.581 (0.043)	0.455 (0.061)
	Post	3.1 ± 0.7	3.5 ± 0.8	3.2 ± 1.0			
Legs (kg)	Pre	9.4 ± 3.2	9.5 ± 2.2	8.8 ± 2.7	0.062 (0.132)	0.894 (0.009)	**0.016** (0.283)^*^
	Post	9.1 ± 3.3	9.3 ± 2.3	9.0 ± 3.0			
Trunk (kg)	Pre	19.2 ± 4.6	20.1 ± 4.5	19.4 ± 3.8	**<0.0001** (0.425)	0.877 (0.010)	0.186 (0.126)
	Post	18.2 ± 4.5	19.4 ± 4.5	19.1 ± 4.4			

*Significant P-value (<0.05) is displayed in bold*.

* *Milk significantly different to both Control and Oat/Potato (Tukey post-hoc analysis). ANOVA, analysis of variance; _p_*η^2^*, partial Eta-squared*.

### Health Markers

There was no effect of the training and drink interventions on any of the measured fasting blood lipids; total cholesterol (tendency, *P* < 0.07), HDL, LDL or triglycerides (Table 7). There were also no significant effects of the training and drink interventions on immune markers TNF-alpha, IL-6 and hs-CRP or fasting glucose, insulin or insulin resistance (HOMA-IR). There was a significant group difference in IL-6, where the Milk group had lower values than Control. This difference was however present already at pre-intervention and cannot be attributed to the test drink. When combining the groups ingesting protein and carbohydrate combinations (Oat/Potato and Milk, *n* = 18) there was a tendency (time × group interaction; *P* = 0.08, _p_η^2^ = 0.12) to lower HOMA-IR after the intervention compared to the Carbohydrate control group (*n* = 10).

**Table 7:** Fasting blood measurements of lipid, glucose and immune metabolism before and after intervention.

		**Control (*n* = 10)**	**Oat/Potato (*n* = 8)**	**Milk (*n* = 10)**	**Two-way ANOVA *P*-value (_p_η^2^)**		
		**Mean ± SD**	**Mean ± SD**	**Mean ± SD**	**Time**	**Group**	**Interaction**
Total Cholesterol (mM)	Pre	5.1 ± 1.5	5.6 ± 0.8	5.0 ± 1.1	0.060 (0.145)	0.584 (0.046)	0.706 (0.030)
	Post	4.8 ± 1.0	5.3 ± 0.9	4.9 ± 1.0			
HDL (mM)	Pre	1.3 ± 0.2	1.4 ± 0.3	1.2 ± 0.3	0.855 (0.001)	0.193 (0.133)	0.203 (0.129)
	Post	1.4 ± 0.2	1.4 ± 0.2	1.1 ± 0.2			
LDL (mM)	Pre	3.5 ± 1.4	3.8 ± 0.9	3.6 ± 1.0	0.114 (0.105)	0.789 (0.020)	0.667 (0.035)
	Post	3.2 ± 1.4	3.6 ± 0.9	3.4 ± 0.9			
Triglycerides (mM)	Pre	1.2 ± 0.6	1.5 ± 0.9	1.2 ± 0.2	0.186 (0.075)	0.898 (0.009)	0.478 (0.062)
	Post	1.4 ± 0.8	1.4 ± 0.7	1.5 ± 0.4			
Glucose (mM)	Pre	5.5 ± 0.3	5.6 ± 0.5	5.4 ± 0.3	0.892 (0.001)	0.308 (0.097)	0.454 (0.066)
	Post	5.7 ± 0.3	5.5 ± 0.3	5.3 ± 0.4			
Insulin (mU/L)	Pre	11.2 ± 4.5	13.0 ± 10.3	9.3 ± 4.4	0.062 (0.143)	0.251 (0.113)	0.110 (0.175)
	Post	12.0 ± 5.8	9.2 ± 4.4	7.2 ± 2.9			
HOMA-IR	Pre	2.8 ± 1.1	3.2 ± 2.5	2.2 ± 1.1	0.091 (0.119)	0.190 (0.343)	0.104 (0.179)
	Post	3.0 ± 1.5	2.3 ± 0.9	1.7 ± 0.7			
CRP (mg/L)	Pre	1.7 ± 1.6	1.7 ± 1.8	1.9 ± 2.6	0.386 (0.033)	0.119 (0.169)	0.857 (0.013)
	Post	1.1 ± 0.7	1.7 ± 2.5	2.3 ± 2.5			
TNFα (pg/mL)	Pre	2.0 ± 0.4	2.0 ± 0.7	2.0 ± 0.6	0.651 (0.009)	0.774 (0.022)	0.718 (0.028)
	Post	1.9 ± 0.8	2.2 ± 0.8	2.2 ± 0.4			
IL-6 (pg/mL)	Pre	0.60 ± 0.20	0.59 ± 0.28	0.40 ± 0.18	0.095 (0.117)	**0.041** (0.243)^*^	0.801 (0.019)
	Post	0.52 ± 0.33	0.42 ± 0.19	0.31 ± 0.12			

*P-value (<0.05) is displayed in bold*.

* *Milk significantly different to Control (Tukey post-hoc analysis). ANOVA, analysis of variance; _p_*η^2^*, partial Eta-squared*.

Although high in BMI, many participants had clinical blood values within the normal range at the start of the intervention. Since one of the aims of the study was to investigate health improvements, a sub-group analysis was performed of subjects outside of the healthy range in order to detect improvements. Out of the 28 participants, using the definition from WHO 1999 guidelines (38), one subject had impaired fasting glucose (IFG; > 6.1 mM) and one had elevated insulin levels (>25 mU/L). Five participants had inflammation marker hs-CRP > 3 mg/L, an indication of high cardiovascular risk according to American Heart Association (39), and using the definition for dyslipidemia from the European society of cardiology (40) thirteen participants had elevated total cholesterol (>5.0 mM), three had low HDL (<1.0 mM), eighteen had elevated LDL (>3 mM) and four had elevated TG (>1.7 mM). When comparing participants with starting cholesterol or LDL values within to those outside of the normal range, no significant two-way interaction was found.

## Discussion

This study showed that in untrained, weight stable men with overweight or obesity performing a six weeks endurance training program, neither milk or oat/potato-based protein supplementation in combination with exercise further improved ${\overset{˙}{\text{V}}\text{O}}_{2\text{peak}}$, performance, recovery or mitochondrial, glycolytic or oxidative skeletal muscle proteins, compared to the Carbohydrate control group. No intervention effect was observed in parameters associated with increased risk of disease, such as fasting blood glucose, insulin, lipids or markers of immune function. For body composition, the intervention decreased total fat mass and increased total lean mass, respectively, with no significant differences between groups. However, the Oat/Potato protein and Carbohydrate control groups had a larger leg fat mass loss compared to the Milk protein group.

Few studies have investigated effects of protein plus carbohydrate vs. carbohydrate only supplementation in conjunction with aerobic exercise on training adaptations such as ${\overset{˙}{\text{V}}\text{O}}_{2\text{max}}$, skeletal muscular measurements and markers of muscle damage, and prior results have been mixed (12–18). These discrepancies are most likely attributable to methodological differences between studies, including length of training intervention, exercise modality and intensity, participants age and training status, and protein supplementation dosage (20–60 g). In a study with similar set-up as the present investigation, Ferguson-Stegall and colleagues (12) found a significant difference in ${\overset{˙}{\text{V}}\text{O}}_{2\text{max}}$ improvement, but no significant difference in CS and SDH activity increase in normal weight untrained participants following protein-carbohydrate supplementation compared to a placebo control and a carbohydrate only drink. Regarding the mechanism underpinning the cardiovascular improvement, they suggested that an increase in plasma volume could have been achieved by a greater increase in plasma albumin content, but did not measure these variables. However, in line with this assumption, Goto et al. (41) reported that five consecutive days of aerobic training in young men, i.e., the same weekly training frequency as in Ferguson-Stegall et al.'s training intervention (12), and a similar post-exercise protein intake dose (0.39 g/kg bodyweight) increased plasma volume and plasma albumin content compared to placebo. Thus, we cannot out rule the possibility that the present study's pre-post protein supplementation protocol (2 × 10 g protein doses supplying 0.2 g/kg body weight) and a lower weekly training frequency was insufficient to further enhance these cardiovascular adaptations compared to the Carbohydrate control group. In contrast to both our and Ferguson-Stegall et al.'s studies, Hansen et al. (16) showed in a 6-week intervention that pre-post exercise ingestion of 0.3 g protein/kg body weight (~40 g protein) significantly increased Cytochrome C protein content compared to isocaloric intake of carbohydrates in trained runners but with no difference in ${\overset{˙}{\text{V}}\text{O}}_{2\text{max}}$. Several other mitochondrial proteins including SDHA, COX-IV, VDAC and HSP60 followed a similar, although non-significant, pattern. However, the primary aim of the study by Hansen et al. (16) was to detect differences in mitochondrial adaptations and was not powered to detect differences in performance outcomes, and it is difficult to translate these results to populations with lower training status such as our cohort. Other studies on endurance training have shown that adding protein to carbohydrate drinks in connection to training suppressed markers of muscle damage 12–24 h post-exercise or improved both performance and recovery (42–44). These studies were generally performed on well-trained athletes during intense and prolonged training. The present study focused on untrained men with overweight/obesity and the possible effect of protein supplementation on training adaptations. The overall improvements in aerobic capacity, enzyme activities and muscle protein levels were in line with previous training interventions of similar design in normal weight or overweight untrained participants (45, 46), but we saw no difference between groups in aerobic or performance improvement. Some changes in skeletal muscular measurements such as increases in HAD and CS activity and GLUT4, SDHA and PDH expression reached significance in the whole population combined. Thus, although we did not include a non-exercising control group, the results indicates that training *per se* is sufficient to induce skeletal muscle adaptations, and that supplementation of either oat/potato- or milk-based protein to carbohydrate drinks had no further impact on these adaptations.

Previous research (47) has shown that higher fat mass negatively influences some domains of physical performance and overall functioning, while lean mass is important relative to amount of body fat. For body composition, the present intervention decreased total fat mass and increased total lean mass, respectively. The only significant interaction effect observed was in leg fat mass, with a ~3% decrease in both Oat/Potato and Carbohydrate control groups and a (non-significant) ~2% increase in the Milk group. Though the explanation for the difference in fat mass loss is not clear, it has previously been demonstrated that post-endurance exercise ingestion of milk protein-carbohydrate drinks was more effective than carbohydrate-only drinks in modulating the activation of key intracellular signaling stimulus for protein synthesis (48, 49). In the present study we found no significant differences in total or regional lean mass accrual between the Carbohydrate control or Protein groups, which is contrary to the results from a 10-week training intervention in endurance athletes, where a daily dose of 20 g protein from a beef-whey blend significantly increased lower body lean mass and tended to increase total lean mass compared to a carbohydrate control (17). It is possible that the present pre-post protein supplementation protocol with 2 × 10 g protein doses separated by 75–90 min in conjunction with exercise in our cohort, providing a total of ~0.2 g protein/kg body weight, might have been insufficient to increase myofibrillar protein synthesis/lean mass accrual compared to the carbohydrate control/aerobic exercise stimulus alone. Recent dose-response research suggests that a slightly higher dose of protein (30 g, ~0.4 g/kg body weight) is required to maximize myofibrillar protein synthesis after a single bout of endurance exercise in healthy men (50). On the other hand, Breen et al. (51) demonstrated that 20 g protein plus carbohydrate post-exercise supplementation increased myofibrillar protein synthesis by 35% compared to a carbohydrate control after a bout of endurance cycling exercise in well-trained men. Again, it is important to note that the present study involved untrained men with overweight/obesity and the interactive effect of exercise (resistance exercise) plus protein feeding on protein synthesis has been shown to be reduced with obesity (21).

Although body composition changed, our participants were kept weight stable for the duration of the intervention. Previous research (52) shows body weight impacts on metabolic markers so it is not unsurprising that there were no changes in blood metabolic markers of inflammation, lipid metabolism or glucose metabolism in our study. A great number of studies link exercise training to a reduced inflammatory state in overweight and obese participants, though the effect is generally seen in interventions longer than 8 weeks and in conjunction with weight loss (53). The lack of change in metabolic markers in our study might be explained by the relatively short duration of the intervention and the fact that the participants generally started from a healthy metabolic status. In line with our results, no changes in lipid parameters or TNFα were observed following 8-weeks of moderate endurance exercise in participants with similar physical characteristics and baseline metabolic values as in the present study (54). In that study, however, CRP and IL-6 decreased. Further, while previous research has shown beneficial effects of increased oat β-glucan intake (≥2.5 g/day, range 2–12 weeks) on blood lipids (e.g., reductions in total cholesterol and LDL), glycemic control and insulin sensitivity (55, 56), we found no difference in these parameters in the Oat/Potato treatment compared to Milk or Control groups. This is not surprising considering the lower β-glucan dose in the present study where 3 g per training session equaled 1.3–1.7 g daily, and that individuals with hypercholesterolemia and/or type 2 diabetes mellitus are more likely to benefit from additional β-glucan intake compared with the relatively healthy volunteers included in our study (56, 57). For glucose metabolism, although not significant, there was a tendency for combined groups ingesting protein and carbohydrate combinations (Oat/Potato and Milk) to lower HOMA-IR over the intervention compared to the Carbohydrate only group. Previous research has shown reduced insulin resistance in obese and overweight subjects combining exercise and increased protein intake, albeit in conjunction with weight loss (58). The results warrant further research into benefits of protein supplementation in insulin resistant subjects, with or without weight-loss.

Although the present study has several strengths including high compliance, supervised training sessions and drink intake, and a controlled 36-h dietary intake during the endurance tests and TTE trials, limitations must be considered. We did not control for daily protein intake during the 6-week intervention period, and the 3-day food record collected at baseline demonstrated a low protein intake in all groups (0.8–0.9 g protein/kg body weight/day; Supplementary Table), but the participants were instructed to maintain their normal diet throughout the study. Due to dropouts and recruitment difficulties, the individual groups were relatively small. This provides a challenge to detect small between-group differences. With such large general training effects in untrained subjects, a potential additional effect of protein supplementation might be masked. However, all data points toward the same conclusion with large training effects without differences between groups. Though the protein content was matched between Oat/Potato and Milk, oat naturally contains fiber (β-glucans), and its possible interaction with protein supplementation is unknown. Furthermore, for calorie balancing purposes the control drink was higher in fat content (equal to an additional 6 g fat/session).

## Conclusions

In conclusion, in untrained men with overweight or obesity, supplementation of plant- or animal-based protein-carbohydrate drinks in connection with aerobic exercise training three times per week for 6 weeks resulted in no further training adaptations (aerobic capacity or upregulation in a number of skeletal muscle proteins) compared to a isocaloric carbohydrate control drink. Some differences in body composition were detected; leg fat mass loss was higher in the Oat/Potato group compared to the Milk group, indicating that plant-based protein supplementation might have differential effects on body composition compared to an animal supplement despite similar amino acid compositions. Furthermore, irrespective of drink treatment, no effect on the lipid profile, immune markers, fasting glucose, insulin or HOMA-IR was observed. The results of the current study therefore suggest that regular supplementation of 2 × 10 g of either plant- or animal-based protein in connection with moderately intense aerobic exercise have no additional effect compared to carbohydrate feeding in untrained exercisers with normal metabolic status. Future studies investigating health markers after exercise training with supplementation of different protein sources would be of high interest in participants with metabolic syndrome or other metabolic disturbances.

## Data Availability Statement

The raw data supporting the conclusions of this article will be made available by the authors, without undue reservation.

## Ethics Statement

The studies involving human participants were reviewed and approved by Local ethical committee at the University of Gothenburg (Dnr.571-14). The patients/participants provided their written informed consent to participate in this study.

## Author Contributions

UA-H, KM, and SP: conceptualization, wrote – original draft preparation and visualization. FE, BE, CH, DS, SW, NJ, and SP: methodology. UA-H and SP: formal analysis. NJ and KM: resources. FE, SP, DS, SW, and UA-H: data curation. UA-H, KM, NJ, BE, and SP: wrote – review and editing. KM: project administration. UA-H and KM: funding acquisition. All authors contributed to the article and approved the submitted version.

## Conflict of Interest

The authors declare that the research was conducted in the absence of any commercial or financial relationships that could be construed as a potential conflict of interest.
