# Supplementary figures and images for: Six Weeks of Aerobic Exercise in Untrained Men With Overweight/Obesity Improved Training Adaptations, Performance and Body Composition Independent of Oat/Potato or Milk Based Protein-Carbohydrate Drink Supplementation
Source: Front Nutr. 2021 Feb 15;8:617344. doi: 10.3389/fnut.2021.617344 (PMC7917245; doi:10.3389/fnut.2021.617344)

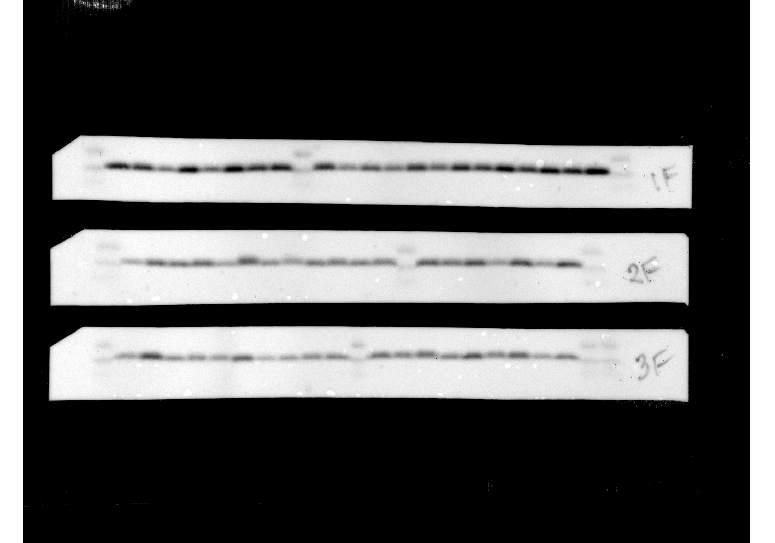

Supplement: Supplementary file 1 [file Presentation_1.ZIP › Original image CytC 4.jpg]

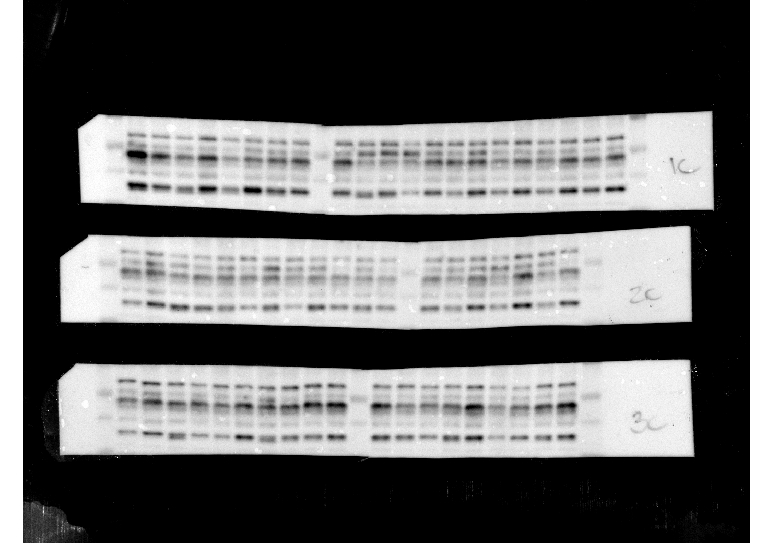

Supplement: Supplementary file 1 [file Presentation_1.ZIP › Original image GLUT 4.jpg]

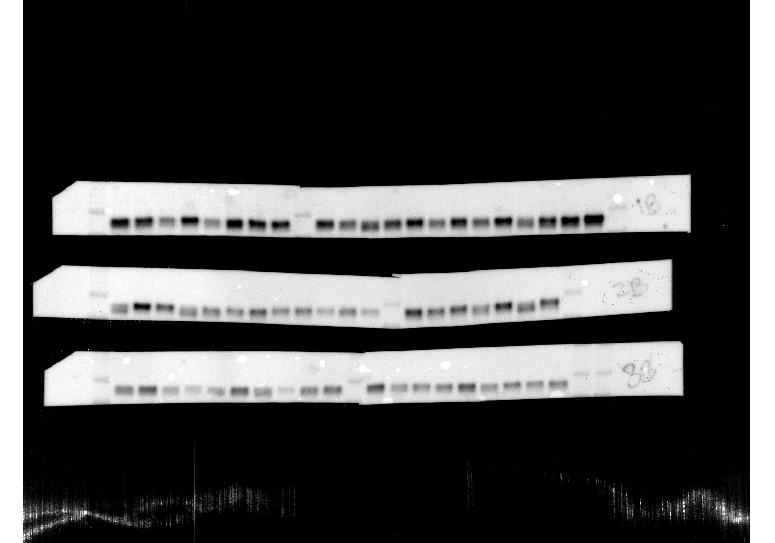

Supplement: Supplementary file 1 [file Presentation_1.ZIP › Original image GS.jpg]

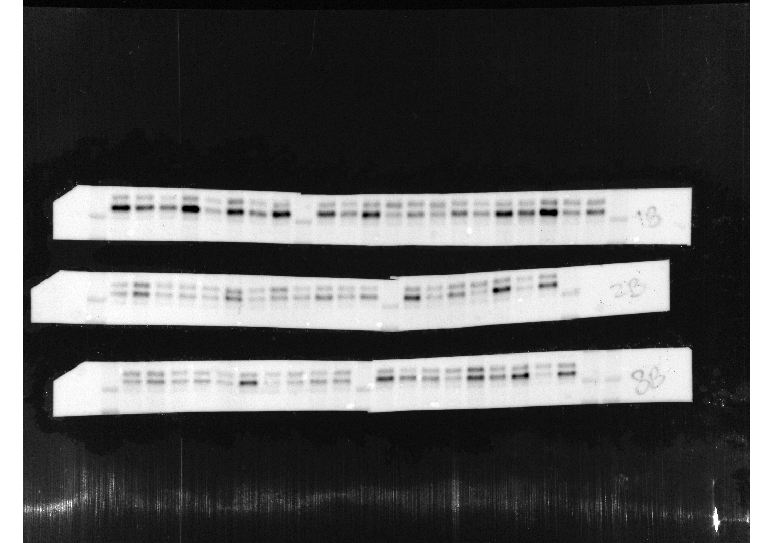

Supplement: Supplementary file 1 [file Presentation_1.ZIP › Original image HK II.jpg]

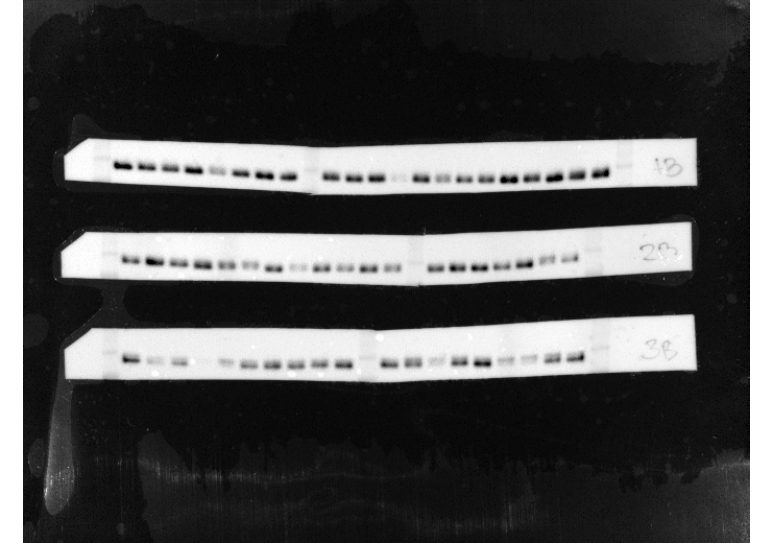

Supplement: Supplementary file 1 [file Presentation_1.ZIP › Original image IR.jpg]

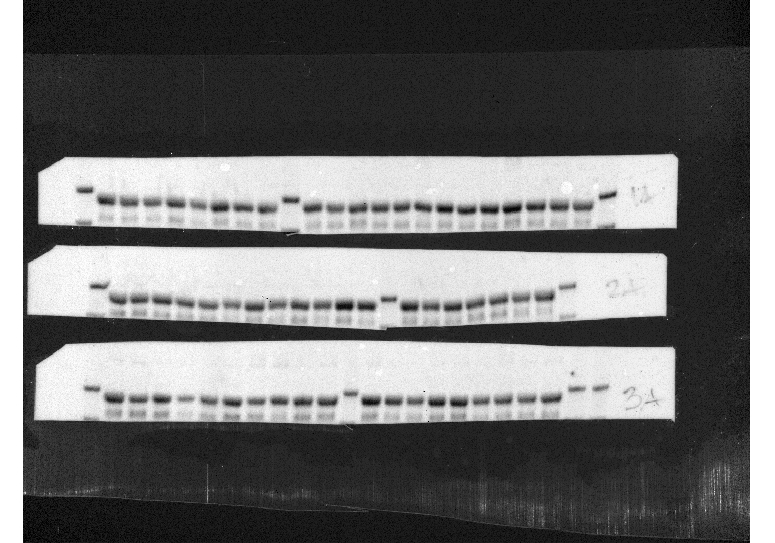

Supplement: Supplementary file 1 [file Presentation_1.ZIP › Original image IRS1.jpg]

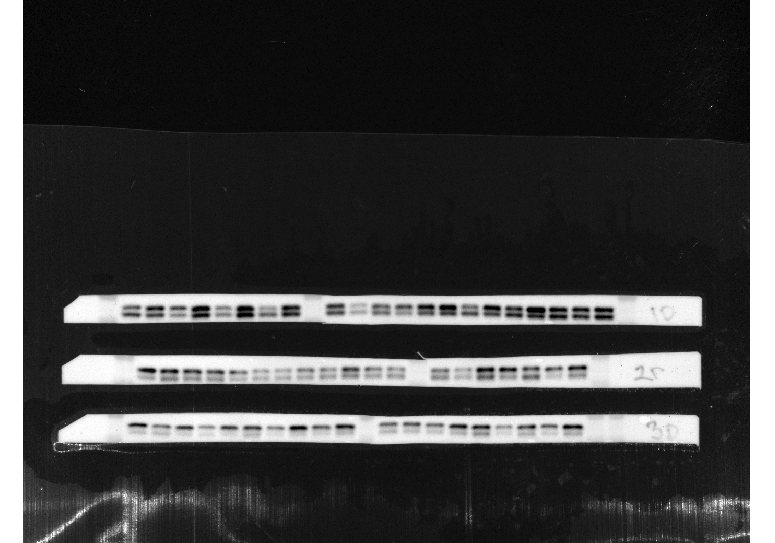

Supplement: Supplementary file 1 [file Presentation_1.ZIP › Original image PDH.jpg]

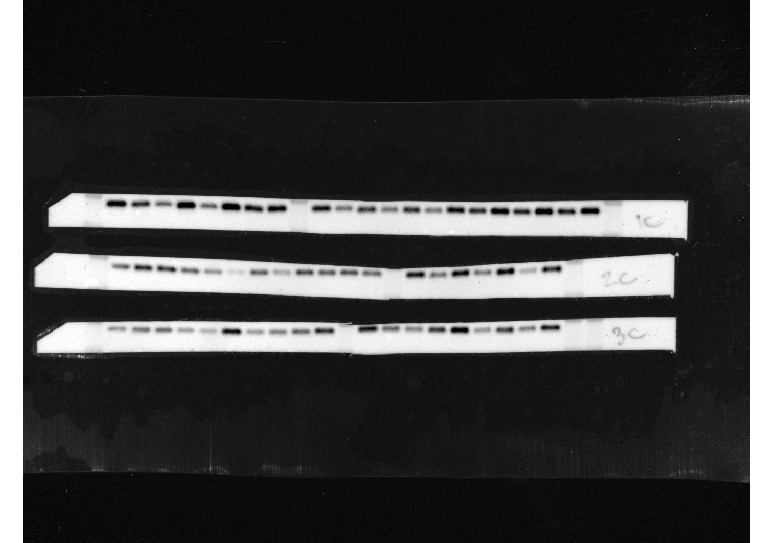

Supplement: Supplementary file 1 [file Presentation_1.ZIP › Original image SDHA.jpg]
