# Supplementary material for: Six Weeks of Aerobic Exercise in Untrained Men With Overweight/Obesity Improved Training Adaptations, Performance and Body Composition Independent of Oat/Potato or Milk Based Protein-Carbohydrate Drink Supplementation
Source: Front Nutr. 2021 Feb 15;8:617344. doi: 10.3389/fnut.2021.617344 (PMC7917245; doi:10.3389/fnut.2021.617344)
Supplement: Supplementary file 2 [file Table_1.DOCX]

Supplementary Material

# Supplementary Table. Baseline dietary intake from 3-day food records.

|  | Control  (n=10)  Mean ± SD | | Oat/Potato  (n=8)  Mean ± SD | Milk  (n=10)  Mean ± SD | Control  vs Oat/Pot | P  Control  vs Milk | Oat/Pot  vs Milk |
| --- | --- | --- | --- | --- | --- | --- | --- |
| Energy intake (kcal) | | 2409 ± 739 | 2221 ± 545 | 2111 ± 407 | 0.625 | 0.410 | 0.719 |
| Protein (g) | | 87 ± 20 | 83 ± 18 | 102 ± 25 | 0.680 | 0.249 | 0.169 |
| Protein (g/kg BM) | | 0.9 ± 0.2 | 0.8 ± 0.2 | 0.9 ± 0.2 | 0.616 | 0.685 | 0.359 |
| Carbohydrate (g) | | 245 ± 123 | 233 ± 67 | 206 ± 51 | 0.839 | 0.497 | 0.482 |
| Fat (g) | | 101 ± 39 | 99 ± 39 | 88 ± 23 | 0.904 | 0.497 | 0.607 |
| *whereof* | |  |  |  |  |  |  |
| Polyunsaturated (%) | | 5.1 ± 1.8 | 6.0 ± 1.9 | 5.1 ± 1.0 | 0.416 | 0.973 | 0.356 |
| Monounsaturated (%) | | 13.4 ± 3.5 | 13.5 ± 3.1 | 14.4 ± 2.0 | 0.956 | 0.544 | 0.565 |
| Saturated (%) | | 15.0 ± 3.5 | 15.8 ± 5.2 | 14.0 ± 1.6 | 0.694 | 0.703 | 0.466 |
